# A Dual-Side Synergistic LoRA Framework for Full-Chain Fine-Tuning of Qwen2.5-VL for Plant Disease Diagnosis

**DOI:** 10.3390/plants15131932

**Published:** 2026-06-23

**Authors:** Zhengyan Zhang, Quan Feng

**Affiliations:** College of Mechanical and Electrical Engineering, Gansu Agricultural University, Lanzhou 730070, China; zhangzheny@gsau.edu.cn

**Keywords:** plant disease diagnosis, dual-side LoRA, full-chain fine-tuning, multimodal large language model, Qwen2.5-VL

## Abstract

The emergence of multimodal large language models (MLLMs) is opening a new avenue for explainable and interactive intelligent diagnosis in agriculture. However, generic MLLMs still face two major obstacles in plant disease recognition—insufficient fine-grained visual perception and misalignment between visual and linguistic features—which jointly limit diagnostic accuracy. To address these issues, we propose a Qwen2.5-VL-based full-chain fine-tuning framework termed dual-side synergistic low-rank adaptation. Unlike the mainstream paradigm that freezes the vision encoder, our method injects trainable LoRA adapters into both the vision encoder and the large language model, while establishing end-to-end gradient backpropagation across the entire multimodal pipeline. By using the supervision signal from autoregressive text generation (text-supervised visual learning), the framework directly drives deep optimization of visual representations, thereby enabling coordinated alignment between pixel-level perception and semantic-level understanding. We trained Qwen over CDDM and conducted in-domain (CDDM) and cross-domain (PlantVillage) experiments. The results show that the proposed 7B-parameter model achieves 98.8 and 96.0% diagnostic accuracy under in-domain and cross-domain scenarios, respectively. The recognition accuracy of Qwen in the case of cross-domain only decreases slightly, which demonstrates that the MLLM trained by our method exhibits excellent cross-domain recognition capability. This indicates that our method can significantly improve the robustness and generalization ability of MLLM in complex agricultural scenarios.

## 1. Introduction

As the world population continues to grow, the tension between food demand and supply is becoming increasingly acute, resulting in global agricultural production being under unprecedented pressure. Worldwide, plant diseases usually reduce crop yields by 20–40% which exacerbates the food supply crisis. According to FAO estimates, plant diseases cost the global economy around USD 220 billion annually [1]. For major staple crops, the average loss rates are approximately 30.3% for rice (*Oryza sativa* L.), 22.6% for maize (*Zea mays* L.), 21.5% for wheat (*Triticum aestivum* L.), and 17.2% for potato (*Solanum tuberosum* L.) [2]. Accurate identification and detection of plant diseases are therefore both necessary and urgent. Traditional diagnosis relies primarily on manual assessment by plant protection experts [3]. Although such assessment can be highly accurate, it is time-consuming, costly, subjective, and heavily dependent on specialized human expertise, making large-scale deployment difficult, particularly in remote areas of developing countries. For farmers, precise disease identification enables targeted pesticide application, improves chemical-use efficiency, and reduces environmental pollution and ecological damage. Consequently, automating plant disease diagnosis with computer vision techniques has become a central objective in smart agriculture [4].

Since 2015, deep learning has become the dominant technical route for plant disease diagnosis [5], driving the transition from handcrafted feature extractors and shallow classifiers to efficient end-to-end learning paradigms [6]. Recent review articles on plant disease recognition [7,8,9,10] show that convolutional neural networks (CNNs) and vision transformers (ViTs) have emerged as the two principal methodological paradigms in this field, with CNNs occupying the de facto dominant position for many years because of their strong automatic feature extraction capability. Representative architectures such as ResNet [11], DenseNet [12], and EfficientNet [13] can effectively capture lesion color, texture, and shape features and have achieved near-saturated classification performance under controlled acquisition conditions. For example, Mohanty et al. [14] reported 99.35% accuracy on a held-out PlantVillage [15], and Ferentinos et al. [16] achieved a maximum recognition rate of 99.53% in a multi-crop, multi-disease classification task. These results demonstrated the feasibility of high-accuracy image-based diagnosis under controlled imaging conditions and provided an important performance benchmark for subsequent studies. Nevertheless, although CNNs exhibit strong local feature extraction capability, their inherent local inductive bias and limited effective receptive field restrict their ability to capture global contextual relationships. To overcome these limitations, Transformer-based vision models, particularly the Vision Transformer (ViT), have attracted increasing attention in crop disease recognition. Built upon the Transformer architecture [17] and first introduced for image recognition by Dosovitskiy et al. [18], ViT has shown promising performance in crop disease identification [19]. Unlike CNNs, which primarily emphasize local pattern extraction, ViTs model images through self-attention and are therefore better suited to capturing long-range dependencies and global semantic information, making them a potentially effective alternative for disease recognition in field images with complex backgrounds, occlusion, and multi-scale symptoms. Beyond predictive accuracy alone, recent agricultural image-analysis studies have also emphasized efficiency and practical usability as important evaluation dimensions. For example, Parlak Sönmez and Kılıç evaluated deep learning-based agricultural image classification from the perspectives of both efficiency and accuracy, suggesting that practical agricultural AI systems should be judged not only by effectiveness, but also by usability and resource cost [20].

A large body of work has nevertheless shown that model generalization under real field conditions remains a critical bottleneck. After reporting 99.35% accuracy on PlantVillage, Mohanty et al. found that accuracy dropped sharply to 31.4% when the same model was tested on web images collected under different imaging conditions [16]. PlantDoc [21], which contains images that better reflect real-world conditions, further highlighted the scarcity of non-laboratory data and the importance of evaluation under realistic scenarios. Even under the challenging PlantVillage-to-PlantDoc cross-domain setting, recent studies have reached only about 68% accuracy [22]. More recent studies have further highlighted that plant disease classification should be evaluated not only by closed-set in-domain accuracy, but also by cross-dataset robustness and deployment-related characteristics. For instance, PlantPathNet incorporated cross-dataset evaluation together with computational efficiency comparison, further reinforcing the importance of robustness and practical applicability in agricultural AI systems [23]. These results indicate that excellent classification performance on controlled datasets does not translate directly into robust field deployment. The core bottlenecks are distribution shift and the insufficient preservation of fine-grained information. Mainstream visual models are typically trained and evaluated at fixed input resolutions; images are uniformly resized to match model requirements, as in Mohanty et al. [14], where images were resized to 256 × 256 pixels. In high-resolution field images, however, early lesions are often small and weak in contrast, so downsampling can erase critical texture and boundary cues. Moreover, uncontrolled factors such as complex backgrounds, illumination variation, and occlusion can further undermine robustness. In addition, image-level classifiers usually output only a disease label and provide little verifiable evidence, making it difficult to integrate contextual information such as environmental conditions and crop management history or to support downstream question answering for disease control. These limitations motivate a transition from single-modality visual perception to multimodal knowledge alignment and cognitive reasoning.

Since 2023, with the maturation of the Transformer architecture and the rapid rise of large language models (LLMs) [24], intelligent agricultural diagnosis has entered a second phase of development, shifting from pure visual perception toward multimodal cognitive reasoning. Multimodal large language models (MLLMs), represented by GPT-4V, LLaVA, and Qwen-VL [25], have gradually been introduced into agriculture and are opening a new paradigm for plant disease recognition [26]. Unlike traditional CNN- or ViT-based discriminative models, which essentially function as closed-set classifiers [27] that map image pixels to predefined labels, MLLMs are capable of semantic understanding and can reason about previously unseen conditions through alignment between a visual encoder and an LLM backbone. This paradigm offers several advantages in agriculture. First, it provides much stronger explainability: MLLMs can not only recognize a disease but also generate detailed symptom descriptions, including color, texture, and affected plant organs, as well as treatment recommendations, thereby moving from “what it is” to “why it is” and “what to do next”. Second, MLLMs exhibit powerful transfer capability. Through instruction tuning [28] and in-context learning [24], they can transfer broad knowledge to agricultural scenarios and show promise for diagnosing rare diseases when large-scale labeled data are limited. Although MLLMs trained on massive datasets can achieve relatively high zero-shot/few-shot and cross-domain recognition accuracy for conventional targets, some studies have shown that their performance in plant disease identification is not optimistic. Liu et al. [29] constructed the GrowLI benchmark with 137,000 images and systematically evaluated Qwen-VL-Chat [30] and LLaVA-v1.5-7B [31] on 30 diseases. Under a zero-shot setting, Qwen-VL-Chat achieved only 28.40% diagnostic accuracy, whereas LLaVA-v1.5-7B achieved only 24.50%, revealing severe hallucination when distinguishing visually similar symptoms, such as early blight versus nutrient deficiency. These observations show that generic pretrained models do not inherently possess agricultural expertise, making domain-specific fine-tuning essential. To improve accuracy, a growing line of work has focused on agricultural adaptation of MLLMs through instruction tuning. Some studies, such as AgroGPT [32], start from a general multimodal model and build agricultural instruction datasets to obtain conversational disease identification and consultation ability. Others, such as PlantVillageVQA [33], construct visual question answering benchmarks to evaluate the model’s capability along the diagnostic chain, including recognition, evidence localization, and causal or counterfactual reasoning. Additional studies have proposed dedicated multimodal large-model frameworks for crop disease recognition, such as LLMI-CDP [34], improved anomaly detection reliability with vision-language models [35], or enhanced recognition through cross-modal fusion of visual features and disease-description text generated or encoded by a VLM [36]. Systematic evaluations of closed-source or general MLLMs, such as GPT-4o, likewise show that zero-shot performance is substantially weaker than that of task-adapted models, whereas few-shot or progressive fine-tuning can produce significant gains [37]. Wang et al. [38] proposed Agri-LLaVA, which uses a high-quality “image–symptom description–treatment dialogue” instruction dataset to fully fine-tune LLaVA, improving disease recognition accuracy under complex field backgrounds by more than 20% while also enabling multi-turn diagnostic dialogue. Similarly, AgriGPT-VL [39] introduced curriculum learning, allowing the model to learn from simple single-disease cases before progressing to more complex multi-disease co-occurrence scenarios, thereby enhancing sensitivity to subtle pathological features. These advances collectively suggest that post-pretraining adaptation to domain-specific data and tasks remains critical for reliable plant disease diagnosis. At present, most MLLM fine-tuning strategies adopt a frozen vision encoder, updating only the language model while leaving the pretrained visual feature extractor unchanged. Although this strategy reduces memory requirements and preserves generic visual features learned from large datasets, it is suboptimal for fine-grained plant disease recognition. Plant disease diagnosis depends heavily on subtle differences in texture, edge morphology, and color distribution. When the vision encoder is frozen, the model cannot adapt its low-level perceptual patterns to the new domain, so the visual features delivered to the language model may already contain noise or omit critical cues, ultimately limiting robustness under complex conditions.

To overcome these limitations, we propose a dual-side synergistic LoRA fine-tuning framework for Qwen2.5-VL [40]. Unlike conventional strategies that treat the vision encoder as a static feature extractor, our method constructs a fully differentiable full-chain framework. Specifically, while partially unfreezing the vision encoder and language generation module, we inject trainable low-rank adapters (LoRA) into the multi-head self-attention layers of the vision encoder and the attention layers of the language module. Through this dual-side collaborative mechanism, the vision encoder can actively adjust its feature distribution according to gradients induced by textual instructions, thereby enabling task-driven perception and focusing on key lesion regions already at the pixel-level feature extraction stage. In addition, the dynamic-resolution mechanism and M-RoPE encoding strategy of Qwen2.5-VL allow the model to represent bounding boxes, points, and other spatial features directly at the actual resolution of the input image. This design enables the model to learn scale information intrinsically and better handle images of different resolutions. The multimodal scalable RoPE strategy begins encoding from the image center and arranges textual encoding along the grid diagonal, thereby improving image-resolution scalability and image–text alignment. As a result, high-resolution texture cues in the original images can be retained more completely, further strengthening plant disease diagnosis.

At present, most MLLM fine-tuning strategies adopt a frozen vision encoder [41], typically updating only the language model while leaving the pretrained visual feature extractor unchanged. More broadly, existing LoRA-based or parameter-efficient multimodal tuning methods are primarily designed for computational efficiency, and they generally follow one of two paradigms: adapting only the language model, or preserving frozen visual representations with lightweight cross-modal bridging. Although such strategies reduce memory requirements and preserve generic visual features learned from large-scale datasets [42], they are suboptimal for fine-grained plant disease recognition, where robust diagnosis depends heavily on subtle differences in lesion texture, edge morphology, color variation, and spatial distribution. In these scenarios, freezing the vision encoder restricts the model’s ability to reshape low-level perceptual patterns toward disease-specific cues, so the visual features delivered to the language model may already contain noise [14] or omit critical information, ultimately limiting cross-modal alignment and robustness under complex field conditions [43]. To address this gap, we propose a dual-side synergistic LoRA framework for full-chain fine-tuning of Qwen2.5-VL. Unlike conventional strategies that treat the vision encoder as a static feature extractor [44], and unlike previous LoRA-based multimodal methods that mainly emphasize parameter efficiency, our framework jointly injects trainable low-rank adapters (LoRA) [45] into both the vision encoder and the language model, enabling simultaneous adaptation of visual perception and semantic reasoning. More importantly, rather than relying on frozen visual features or separate alignment stages, the proposed framework establishes an end-to-end gradient backpropagation pathway under a unified autoregressive objective, allowing textual supervision to directly refine visual representations and thereby enhancing task-driven lesion perception. In addition, by combining dual-side parameter-efficient adaptation with the dynamic-resolution mechanism and M-RoPE encoding strategy of Qwen2.5-VL [46], the framework preserves high-resolution texture cues more effectively, improves image–text alignment, and is therefore particularly suited to robust plant disease diagnosis in realistic agricultural environments. Therefore, the novelty of our method lies not merely in applying LoRA to two modules, but in establishing a task-oriented multimodal adaptation mechanism that explicitly couples fine-grained visual refinement with language-side reasoning for plant disease diagnosis under domain shift.

## 2. Results

### 2.1. Experimental Environment and Hyperparameter Settings

All experiments were conducted on an AI workstation. The hardware configuration consisted of an Intel i9-14900K processor (Intel Corporation, Santa Clara, CA, USA), one NVIDIA GeForce RTX 4090 GPU (NVIDIA Corporation, Santa Clara, CA, USA) with 48 GB of memory, and 192 GB of system RAM. The software environment ran on 64-bit Windows 11. Model training was implemented with LLaMA-Factory using Python 3.10 (Python Software Foundation, Wilmington, DE, USA) and PyTorch 2.6.0. (PyTorch Foundation, Wilmington, DE, USA).

### 2.2. Training

**Figure 1:**
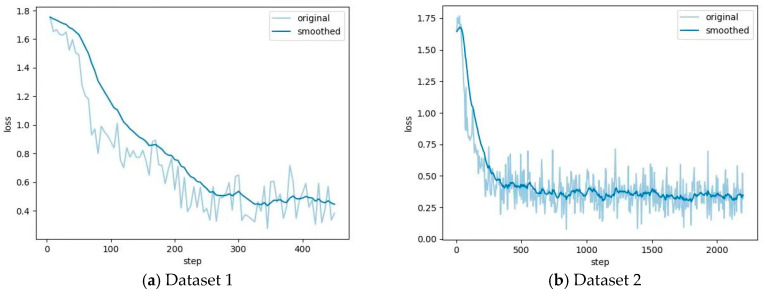

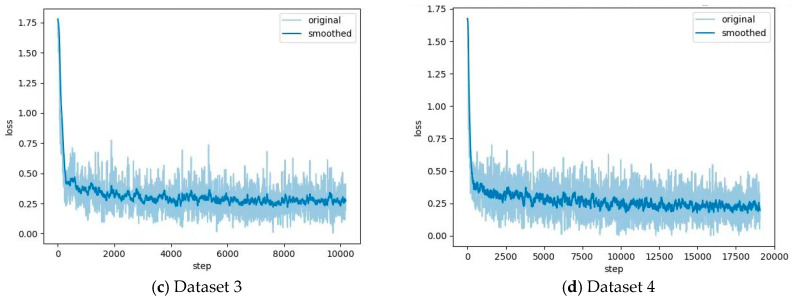
Training loss curves during fine-tuning on four datasets of different scales.

To investigate the influence of training-data scale on model convergence and feature-learning capability, Qwen2.5-VL-7B was fine-tuned using LoRA on four training databases of different scales constructed from the publicly available plant disease dataset CDDM [29], as listed in Section 4.3. Each fine-tuning experiment was conducted on an NVIDIA RTX 4090 GPU (NVIDIA Corporation, Santa Clara, CA, USA), and all training parameters were kept identical across the four runs to ensure a fair comparison. The hyperparameters were set as follows: batch size = 2, number of training epochs = 3, and learning rate = 5 × 10^−5^. The LoRA configuration used a rank of 64 and an alpha value of 128. The loss curves observed during fine-tuning are shown in Figure 1.

The four fine-tuning loss curves, where the light-blue curves denote the raw step-wise loss and the dark-blue curves denote the smoothed trends, reveal a highly consistent pattern. Specifically, the loss decreases rapidly during the early training stage and then gradually enters a plateau. Moreover, with more sufficient training, corresponding to a larger data scale and/or a greater number of training steps, the plateau loss becomes lower, although the marginal improvement progressively diminishes. In the first curve, covering approximately 0–450 steps, the loss rapidly decreases from about 1.7 to approximately 0.45, while still showing a slow downward trend at the end of training. This indicates that the model has initially learned the most salient discriminative cues, such as large-scale color variations and obvious lesion textures, but has not yet fully converged. Meanwhile, the relatively pronounced fluctuations in the raw loss curve suggest insufficient effective sample coverage or high within-batch heterogeneity, which may lead to large single-step gradient noise and thus a more evident zigzag pattern. In the second curve, covering 0–2200 steps, the loss rapidly drops to 0.35–0.40 within the first 300–500 steps and then reaches a relatively stable plateau at around 0.33. This corresponds to a sharp reduction in loss when the training data scale increases from a small to a medium level, indicating that the model has largely captured the high-frequency and easily learnable visual patterns, whereas the remaining patterns are more subtle and difficult to learn, making further loss reduction much slower. In the third and fourth curves, where training is extended to approximately 10,000 and 20,000 steps, respectively, the smoothed loss curves tend to stabilize at around 0.28 and 0.25. Although the loss continues to decrease, its slope becomes increasingly smaller, showing a typical diminishing-returns trend.

**Figure 2:**
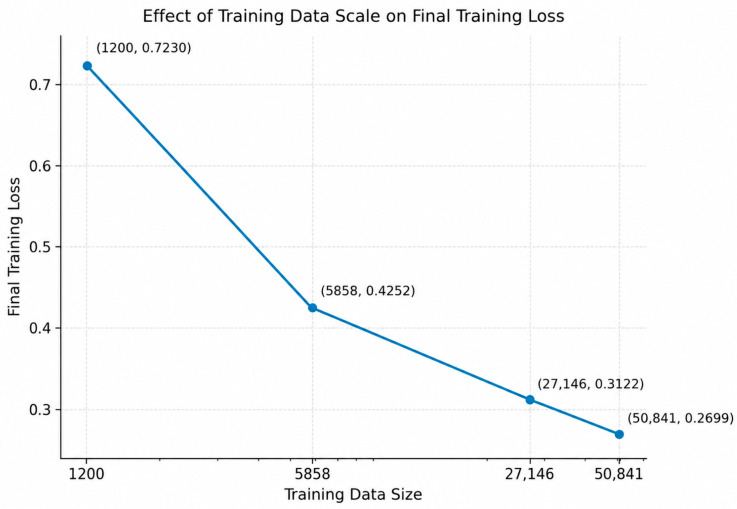
Effect of dataset scale on training loss.

Taken together, the four training curves exhibit a consistent “fast-then-slow” convergence trend. At the early stage of training, the loss decreases rapidly, indicating that the model can quickly learn the primary discriminative features of plant diseases. As shown in Figure 2, when the dataset size increased from 1200 to 5868 samples, the loss dropped sharply from 0.7230 to 0.4252. When the dataset size was further expanded to 27,146 and 50,841 samples, the loss decreased to 0.3122 and 0.2699, respectively. Although the loss continued to decline, the magnitude of improvement became smaller, indicating that training was gradually approaching the best achievable level under the current model and optimization configuration.

### 2.3. In-Domain and Cross-Domain Evaluation of the Proposed Framework

To comprehensively evaluate the performance of the LoRA-adapted Qwen2.5-VL model on plant disease recognition, we designed a dual evaluation protocol consisting of in-domain assessment and cross-domain generalization testing. Top-1 accuracy was used as the core metric throughout. Top-1 accuracy is the proportion of test samples for which the class with the highest predicted probability exactly matches the ground-truth label. This metric directly reflects the discriminative capacity and confidence of the model in fine-grained disease recognition.

**Figure 3:**
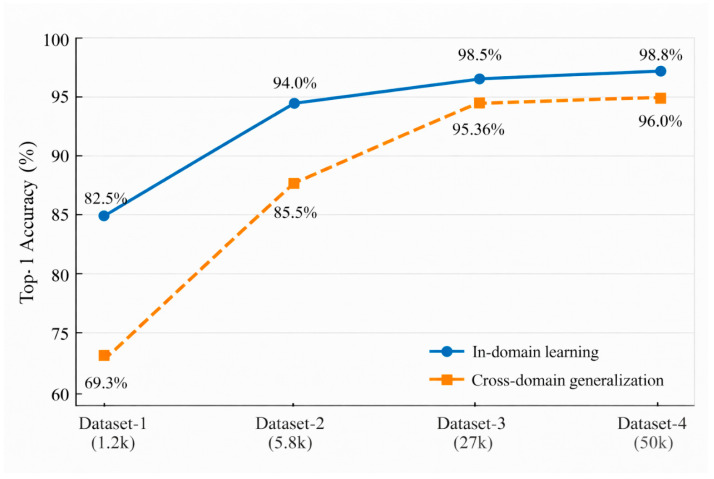
Performance evolution under in-domain and cross-domain evaluation.

We first conducted in-domain testing [47] using an independent held-out test set from CDDM, which is distributionally aligned with the training data. Because the training and test sets are highly consistent in image acquisition environment, class composition, and feature distribution, this experiment primarily evaluates fitting ability and convergence under same-domain conditions. As shown in Figure 3, Top-1 accuracy increased steadily from 82.5% to 98.8% as the training data scale expanded, indicating that the proposed method can effectively learn and represent key disease-related visual features in the dataset. Compared with domain-adapted models reported in [29] with the accuracies of 91.5% and 91.8% for Qwen-VL-Chat-AG and LLaVA-AG, respectively, our model reaches a substantially higher same-domain recognition level.

We then conducted cross-domain testing to further assess robustness under previously unseen conditions. PlantVillage was used as the cross-domain benchmark. Because CDDM contains images acquired in complex field environments, whereas PlantVillage consists primarily of laboratory-background images, the two datasets exhibit a marked domain shift. This setting therefore provides a stringent test of whether the model has learned disease-intrinsic features rather than merely memorizing background statistics.

As shown in Figure 3, cross-domain accuracy also improved progressively as the training-set size increased, reaching a maximum of 96.0%. Even with only 5868 training images—approximately 4% of the complete CDDM dataset—the model already achieved 85.5% accuracy, indicating that the pretrained visual encoder of Qwen2.5-VL provides strong feature extraction capability and can be effectively adapted with limited domain data. As the dataset size increased from 1200 to 50,841 samples, accuracy rose substantially, and performance exceeded 95% once the training set reached 27,146 samples. These results suggest that for domain-specific plant disease recognition, tens of thousands of high-quality multimodal instruction samples may be sufficient to unlock the domain capability of a large multimodal model, without requiring million-scale data.

To provide a more intuitive comparison of the performance differences among multimodal large models in same-domain and cross-domain plant disease recognition tasks, Table 1 summarizes the experimental results of several representative models. Considering that the subsequent discussion refers to the domain-gap phenomenon reported by Mohanty et al. [14], both the same-domain and external-validation results of their best-performing GoogLeNet model are included. The model achieved very high accuracy on the held-out PlantVillage test set, whereas its performance dropped sharply on external validation datasets, highlighting the limited cross-domain robustness of models trained under controlled laboratory-image conditions. It should be noted that Qwen-VL-Chat and LLaVA-v1.5-7B in the table are generic multimodal backbone models without further adaptation using agricultural-domain data; their results are included to reflect the initial performance of general pretrained models in disease recognition scenarios. In contrast, Qwen-VL-Chat-AG and LLaVA-AG are agriculturally adapted models obtained through domain-specific fine-tuning on the CDDM dataset, and their results are cited from the relevant literature. The entry reported as “Our method” corresponds to the best-performing model configuration shown in Figure 3 and represents present the final performance of the proposed method in cross-domain disease recognition.

Compared with existing methods, our analysis emphasizes cross-domain generalization rather than only same-domain accuracy under closed conditions. Previous studies have shown that conventional visual models can achieve very high accuracy within a closed in-domain environment. For example, the best CNN model reported by Mohanty et al. achieved 99.35% accuracy on the PlantVillage in-domain test [14]. However, when tested on external datasets, its accuracy dropped dramatically to 31.40% and 31.69%, indicating that high in-domain accuracy does not necessarily translate into strong cross-domain robustness. Meanwhile, in [29], the base models Qwen-VL-Chat and LLaVA-v1.5-7B achieved only 28.4% and 24.5% crop-disease classification accuracy, respectively, without disease domain adaptation. After domain fine-tuning on CDDM, the accuracies of Qwen-VL-Chat-AG and LLaVA-AG increased to 91.5% and 91.8%, demonstrating that domain adaption can effectively promote the learning of disease-relevant agricultural features. Building on this foundation, our method performs dual-side LoRA adaptation of Qwen2.5-VL on the CDDM50k dataset and further improves cross-domain disease recognition. In cross-domain testing on PlantVillage, the model ultimately achieves a Top-1 accuracy of 96.0%. This result suggests that the proposed framework learns more transferable disease representations rather than shallow pixel-distribution cues tied to the training domain.

### 2.4. Ablation Study and Qualitative Analysis of Model Behavior

To better understand not only the overall performance gain but also its source, we further conducted ablation analysis, macro-level evaluation, local confusion-matrix analysis, and representative case studies.

#### 2.4.1. Ablation Settings and Quantitative Comparison

To quantify the contribution of each trainable branch in the proposed framework, we designed three adaptation settings: language-side LoRA only, vision-side LoRA only, and dual-side LoRA. In the language-only setting, LoRA adapters were injected only into the self-attention layers of the language model, while the vision encoder remained frozen. In the vision-only setting, LoRA adapters were injected only into the MHSA layers of the vision encoder, while the language model remained unchanged. In the dual-side setting, both the vision encoder and the language model were jointly adapted. To ensure a fair comparison, all variants used the same backbone model, training dataset, optimizer settings, LoRA hyperparameters, and evaluation protocol. Specifically, all experiments were conducted on Dataset 4, which contains 50,841 training samples. The hyperparameters were set as follows: batch size = 2, number of training epochs = 3, and learning rate = 5 × 10^−5^. The LoRA configuration used a rank of 64 and an alpha value of 128.

The results are summarized in Table 2. The language-only setting achieved 74.6% Top-1 accuracy in the in-domain evaluation and 52.3% in the cross-domain evaluation, indicating that language-side semantic adaptation alone is insufficient for robust plant disease diagnosis when the vision encoder remains frozen. In contrast, the vision-only setting substantially improved the performance to 87.4% in-domain and 85.5% cross-domain, demonstrating that visual-side adaptation plays a much more direct role in fine-grained lesion perception and cross-domain generalization. Notably, the dual-side setting achieved the best overall performance, reaching 98.8% Top-1 accuracy in-domain and 96.0% cross-domain. Compared with the language-only setting, the dual-side framework improved in-domain and cross-domain Top-1 accuracy by 24.2 and 43.7 percentage points, respectively. Compared with the vision-only setting, the dual-side design still yielded additional gains of 11.4 percentage points in-domain and 10.5 percentage points cross-domain. These results demonstrate that vision-side adaptation and language-side adaptation provide complementary rather than redundant contributions.

Because overall Top-1 accuracy can be influenced by class-frequency imbalance, we further report Macro-Precision, Macro-Recall, and Macro-F1 in the cross-domain evaluation to provide a more comprehensive assessment of model performance. Unlike accuracy, these macro-averaged metrics assign equal weight to each disease category and therefore offer a more balanced evaluation across both high-frequency and low-frequency classes. As shown in Table 2, the proposed dual-side framework achieved 95.4% Macro-Precision, 94.8% Macro-Recall, and 95.1% Macro-F1, all of which were substantially higher than those of the language-only and vision-only settings. These strong macro-level results further confirm that the performance gain of the proposed framework is not merely driven by majority classes. Instead, the dual-side model not only improves overall recognition accuracy, but also maintains a better balance between prediction reliability and class-wise retrieval capability across different disease categories.

#### 2.4.2. Local Confusion-Matrix Analysis of Difficult Categories

**Figure 4:**
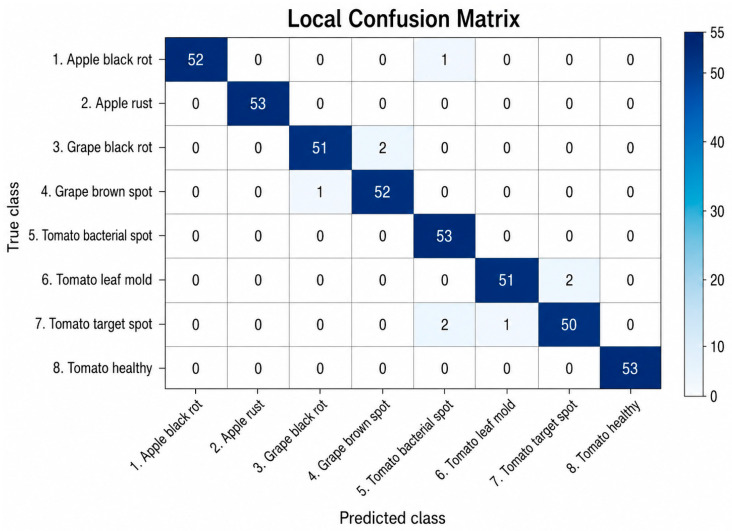
Local confusion matrix of the proposed dual-side LoRA framework on eight representative plant disease categories under cross-domain evaluation.

To further examine class-level prediction behavior, a local confusion matrix was constructed for eight representative disease categories (Figure 4). Most predictions were concentrated along the main diagonal, indicating reliable recognition for the selected classes. Apple (*Malus domestica* Borkh.) rust, Tomato (*Solanum lycopersicum* L.) bacterial spot, and Tomato healthy were classified without error, whereas Apple black rot showed only one misclassification. The remaining errors were mainly observed between visually similar categories, particularly Grape (*Vitis vinifera* L.) black rot versus Grape brown spot, and Tomato leaf mold versus Tomato target spot. Notably, Tomato target spot exhibited the highest local confusion, with misclassifications mainly directed to Tomato bacterial spot and Tomato leaf mold. These results indicate that the residual errors of the proposed framework are primarily associated with highly similar fine-grained symptom patterns, rather than with a general failure of recognition.

#### 2.4.3. Representative Misclassification Examples and Difficult but Correctly Recognized Cases

While the local confusion matrix reveals the overall distribution of confusing category pairs, representative image-level cases provide more intuitive evidence of why such errors occur. Therefore, representative misclassification examples and difficult but correctly recognized cases under cross-domain evaluation were further summarized in Table 3.

The misclassified examples were mainly associated with disease categories sharing highly similar lesion texture, spot morphology, or color distribution. For example, Tomato Target Spot may be confused with Bacterial Leaf Spot when the lesions are small and densely distributed, whereas Grape Black Rot may be confused with Grape Brown Spot when the darker necrotic center is not sufficiently prominent. Likewise, Tomato Leaf Mold may be misclassified as Tomato Target Spot because both diseases can present as brown leaf lesions with similar local texture and spot contours. By contrast, the correctly recognized difficult cases show that the proposed framework is still capable of capturing subtle disease-specific cues under highly confusing visual conditions. For instance, although some Grape Black Rot samples are visually similar to Grape Brown Spot, the model can still recognize the more distinct necrotic center and lesion boundary. Similarly, difficult cases of Tomato Leaf Mold and Tomato Bacterial Spot were still correctly recognized when the model captured differences in lesion distribution, boundary characteristics, and fine-grained texture. Together, these examples further support the conclusion that the remaining errors of the proposed framework are mainly concentrated in highly similar fine-grained disease categories, while its overall visual discrimination capability remains strong in realistic agricultural scenarios.

From the perspective of domain shift, the performance drop from in-domain to cross-domain evaluation was 22.3 percentage points for the language-only setting, 1.9 percentage points for the vision-only setting, and 2.8 percentage points for the dual-side setting. This trend suggests that visual-side adaptation is the dominant factor in improving robustness under domain shift, while language-only adaptation cannot adequately compensate for the lack of disease-specific visual refinement. Although the vision-only model exhibited the smallest performance drop, the dual-side model still delivered the highest absolute accuracy under both in-domain and cross-domain settings. Overall, the ablation study strongly supports our claim that the superiority of the proposed framework arises from the collaborative adaptation of both branches under the full-chain autoregressive objective, rather than from parameter-efficient tuning alone.

### 2.5. Repeated Experiments and Statistical Stability Analysis

To further evaluate the statistical stability of the proposed framework, we repeated the experiment only for the full dual-side LoRA setting (Dual-side, Ours) with five different random seeds, while keeping the model architecture, data split, optimizer settings, and LoRA hyperparameters unchanged. This setting corresponds to the final model reported in this study and represents the core contribution of the proposed method. Since the purpose of the repeated experiment was to verify the reliability and reproducibility of the main reported results, we focused on the complete dual-side framework rather than all ablation variants.

The repeated-experiment results are summarized in Table 4. The five runs yielded in-domain/cross-domain Top-1 accuracies of 98.8%/96.0%, 97.9%/95.2%, 98.7%/95.8%, 98.5%/95.6%, and 98.6%/95.9%, respectively. The averaged results indicate that the proposed framework achieved an in-domain Top-1 accuracy of 98.50 ± 0.35% and a cross-domain Top-1 accuracy of 95.70 ± 0.32%. The relatively small standard deviations indicate that the reported performance of the proposed full model is stable and reproducible. These results suggest that the performance gain of the proposed framework is not dependent on a favorable random initialization but reflects the stability of the dual-side collaborative adaptation mechanism under the current experimental setting.

## 3. Discussion

The results of this study reveal a clear difference between in-domain and cross-domain evaluation, and this difference is highly informative for understanding the generalization ability of plant disease recognition models. In-domain testing, where training and test images come from the same dataset, mainly reflects a model’s ability to fit the statistical characteristics of a specific data source, including image acquisition conditions, annotation style, symptom appearance, background composition, and class distribution. Under this setting, specialized discriminative models such as CNNs often achieve higher accuracy because they are optimized directly for closed-set classification and can effectively exploit dataset-specific visual regularities. However, this strength can become a limitation under cross-domain evaluation. When the target dataset differs in image quality, illumination, leaf pose, disease severity, cultivar appearance, or background clutter, models trained primarily as task-specific classifiers are more likely to rely on spurious or domain-dependent cues, which leads to a sharper performance drop. More broadly, the domain generalization literature has repeatedly shown that high in-domain accuracy does not necessarily translate into robust performance under distribution shift.

Our experiments show that although the fine-tuned MLLMs do not surpass specialized CNN/ViT models on the same dataset (see Table 1), it performs substantially better under cross-domain evaluation. From the perspective of MLLMs, this pattern is reasonable. MLLMs are pretrained on massive image-text pairs and therefore learn semantically aligned representations that are broader and less tied to the visual statistics of any single downstream dataset. Such pretrained models tend to encode higher-level semantic concepts rather than relying exclusively on narrow task-specific textures or color distributions. In the context of plant disease recognition, this means that the model may rely more on semantically meaningful symptom patterns—such as lesion structure, chlorosis, necrosis, mildew-like coverage, or spatial distribution of damage—than on accidental properties of a particular image repository. As a result, even when its same-domain accuracy is not the highest, its recognition ability is more likely to remain stable when transferred to images collected under different cameras, lighting conditions, plant varieties, backgrounds, or disease severity levels.

In addition, from Table 1, we also observed that although MLLMs such as Qwen-VL-Chat and LLaVA-v1.5 perform well on general visual understanding benchmarks, their zero-shot accuracy for cross-domain plant disease diagnosis remains only about 20–30% [29]. This finding highlights a substantial knowledge gap when generic models are directly applied to specialized vertical domains. Generic visual encoders tend to attend to salient objects rather than the fine-grained pathological details that are essential in plant pathology, such as changes in lesion boundary and texture. Accordingly, MLLMs necessitate additional domain-specific fine-tuning to accommodate domain-oriented applications. Table 1 demonstrates that all MLLMs obtain remarkable accuracy enhancements in both in-domain and cross-domain recognition tasks following fine-tuning. However, the extent of accuracy enhancement is evidently closely related to training strategies. After introducing dual-side synergistic LoRA and fine-tuning Qwen2.5-VL-7B, the same-domain disease-recognition accuracy reaches 98.8%, demonstrating competitiveness with specialized conventional models with respect to in-domain testing. More importantly, the improvement is not limited to language adaptation. The training procedure also reshapes the model’s visual attention mechanism, enabling it to isolate disease-relevant cues from complex field backgrounds. This result indicates that end-to-end visual adaptation is indispensable for fine-grained recognition. Furthermore, the Qwen2.5-VL model trained on the CDDM multimodal dataset still achieves 96.0% accuracy on PlantVillage with respect to cross-domain testing, which is far above the performance of generic baselines and exceeds that of prior specialized models. This suggests that the model is not merely memorizing pixel-level distributions from the training set, but is instead learning more transferable disease representations. The advantage of the multimodal framework lies in its simultaneous use of visual information—such as lesion shape, color change, and texture abnormality—and semantic constraints introduced through textual description, thereby establishing stable associations among visual symptoms, language descriptions, and disease categories during training. Prior work has shown that dense textual annotation can provide richer discriminative information than a single class label and that gradients from autoregressive generation can drive the vision encoder toward more semantically discriminative representations. The strong cross-domain performance of Qwen2.5-VL therefore reflects its superior capacity for learning intrinsic disease representations compared with conventional CNN models that primarily fit pixel distributions.

The experimental results further show that the LoRA-adapted 7B model achieves high diagnostic performance while keeping resource consumption within a practically manageable range, suggesting a feasible deployment paradigm for smart agriculture based on cloud-side computation and field-side application. To further evaluate the practical deployability of the proposed framework, we analyzed its computational efficiency on the experimental platform described in Section 2.1. Under the NVIDIA GeForce RTX 4090 GPU with 48 GB memory, the complete training process on dataset 4 required approximately 13.85 h, with a peak GPU memory usage of 28 GB. The final training throughput reached 3.06 samples/s and 0.382 steps/s. The total computational cost was 2.65 × 10^18^ FLOPs, with 56,912,640 input tokens processed during training. In addition, the proposed dual-side LoRA framework trained only 5,575,680 parameters out of 8,297,742,336 total parameters, corresponding to 0.0672% of the full model. These results indicate that the LoRA-adapted 7B framework can achieve strong diagnostic performance while keeping the training cost within a practically acceptable range for cloud-side deployment in smart agriculture. Nevertheless, several limitations still remain. First, although LoRA substantially reduces training cost, the computational demand of a 7B model is still too high for extremely low-power Internet-of-Things sensor nodes. Second, the present datasets mainly cover common diseases, whereas rare diseases on less common crops remain challenging and will likely require a stronger few-shot learning capability in future work. Third, although the current results provide encouraging evidence of robustness under cross-domain evaluation, further validation on an independently collected real-world field dataset would more strongly support the practical applicability of the proposed framework and will be pursued in future work.

## 4. Materials and Methods

### 4.1. Datasets

In this study, two open-source plant disease datasets were used to train and evaluate the proposed model: PlantVillage [15] and the multimodal instruction dataset CDDM [29].

#### 4.1.1. PlantVillage Dataset

**Figure 5:**
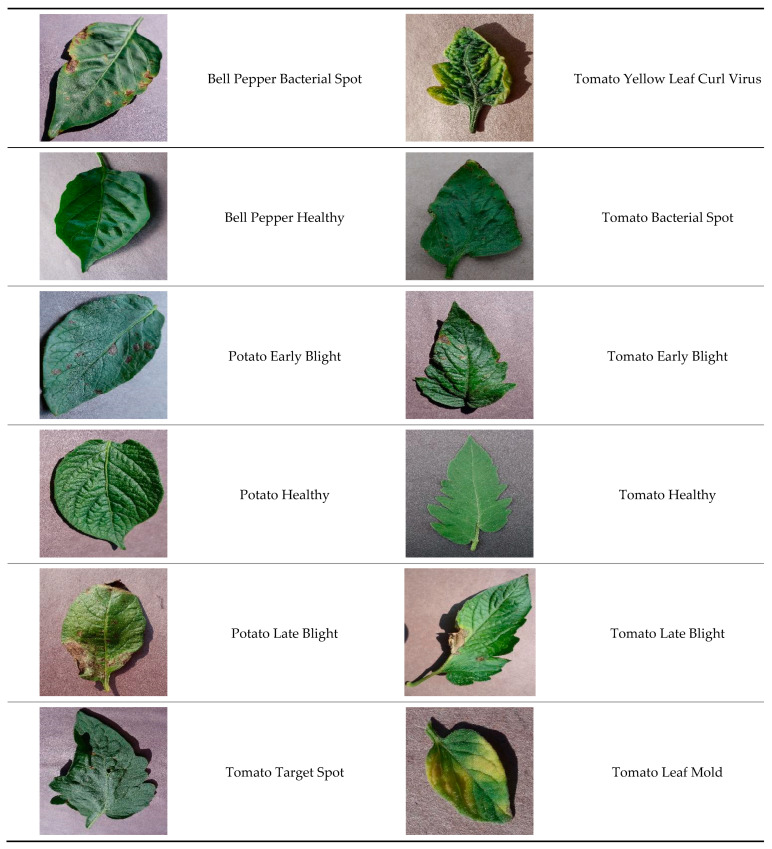
Representative disease examples from the PlantVillage dataset.

PlantVillage contains 54,303 crop disease images covering 14 crops and 38 categories. The images were captured under uniform backgrounds, with leaves prominently centered and disease symptoms clearly visible. All images are provided at a resolution of 256 × 256 pixels. Table 5 lists the crop types and disease categories in PlantVillage, and Figure 5 presents representative disease examples from this dataset.

#### 4.1.2. CDDM Dataset

**Figure 6:**
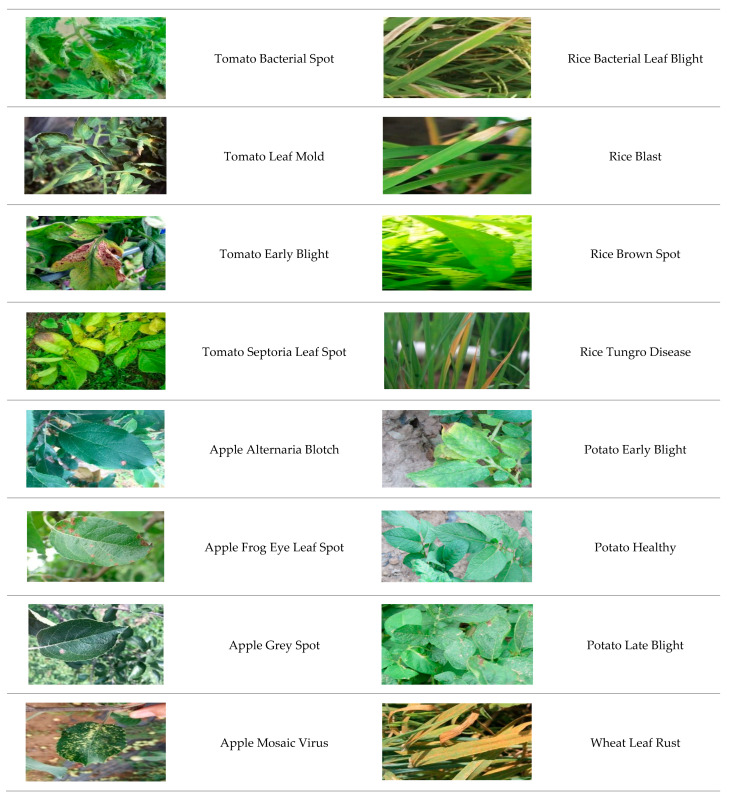
Disease examples from the CDDM dataset.

The second dataset used in this work is the publicly available CDDM crop disease dataset. As summarized in Table 6, CDDM contains 137,000 crop disease images spanning 16 major crops and 60 common disease categories. Figure 6 shows representative examples from the dataset. Unlike PlantVillage, most CDDM images were captured under natural illumination and complex backgrounds. The dataset includes approximately 75,000 field images collected through on-site surveys across multiple farms and orchards, making it substantially more representative of realistic agricultural environments.

### 4.2. Backbone Model: Qwen2.5-VL-7B

**Figure 7:**
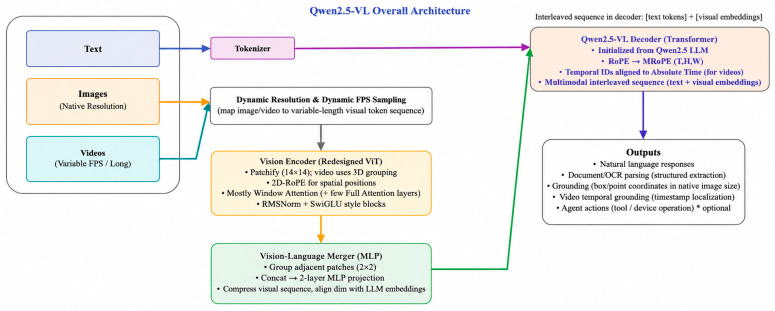
Schematic overview of the Qwen2.5-VL architecture.

Considering the cross-scale feature challenge inherent in plant disease recognition [48]—namely, the need to identify both global leaf structure and fine-grained lesion texture—together with the requirement for complex instruction understanding, we selected Qwen2.5-VL-7B as the multimodal backbone model. According to the Qwen2.5-VL technical report [40], the 7B model compares favorably with state-of-the-art systems such as Claude-3.5-Sonnet-0620, GPT-4o-0513, InternVL2.5, and multiple variants of Qwen2-VL [46]. Figure 7 illustrates the overall architecture of Qwen2.5-VL.

Qwen2.5-VL demonstrates strong general multimodal capability. Its high performance and open-source availability make it a suitable candidate for domain-specific adaptation. More importantly, it is not merely a loose combination of a visual encoder and an LLM. Instead, its superiority over conventional fixed-resolution MLLMs arises from three key architectural strengths.

Native dynamic-resolution perception: in the spatial domain, Qwen2.5-VL can dynamically convert images of different sizes into token sequences of corresponding lengths. This mechanism preserves subtle pathological details in the original high-resolution images and gives the model a microscope-like fine-grained perceptual ability.

Multimodal positional perception based on MRoPE: MRoPE decomposes positional encoding into temporal, height, and width components, enabling the model to capture the relative geometric positions of lesions on leaves more precisely.

Strong instruction-following backbone: on the language-decoding side, Qwen2.5-VL adopts Qwen2.5-7B-Instruct, which has been extensively trained for instruction following. It can not only understand complex disease descriptions in depth, but also generate structured outputs under predefined constraints, making it particularly suitable for fine-grained multimodal instruction tuning and automated downstream processing.

### 4.3. Construction of the Multimodal Instruction Tuning Dataset

It should be noted that class imbalance is a non-negligible characteristic of agricultural disease datasets. In the original CDDM data distribution, several disease categories contain only a limited number of samples, whereas high-frequency categories include substantially more images. If directly used for fine-tuning, such skewed distributions may bias the model toward majority classes and weaken its sensitivity to minority disease categories. Therefore, before constructing the instruction-tuning data, we explicitly considered the effect of class imbalance and adopted a sampling strategy that could alleviate the dominance of high-frequency categories while preserving the available information of low-frequency classes.

To systematically investigate the influence of training-data scale on MLLM performance in a specialized agricultural domain, we built four training subsets from the large-scale CDDM crop disease dataset using stratified balanced sampling [49]. A fixed number of samples was drawn from each disease category, while all samples were retained for categories with insufficient instances. As shown in Table 7, the resulting four subsets contained 1200, 5868, 27,146, and 50,841 samples, respectively, and are hereafter referred to as dataset 1 to dataset 4. To match the supervised fine-tuning (SFT) paradigm of Qwen2.5-VL [50], the sampled raw data were further converted into standard JSONL-formatted image–text instruction pairs. Each sample was organized as a multi-turn dialogue composed of a user instruction and a model response, with three core fields: id (unique identifier), image (image path), and conversations (multi-turn dialogue list). To mimic realistic human–machine interaction, all samples were formatted as dialogues between “User” and “Assistant”, and the image placeholder <image> was embedded in the first user turn so that the model would treat visual input as contextual information for reasoning.

**Figure 8:**
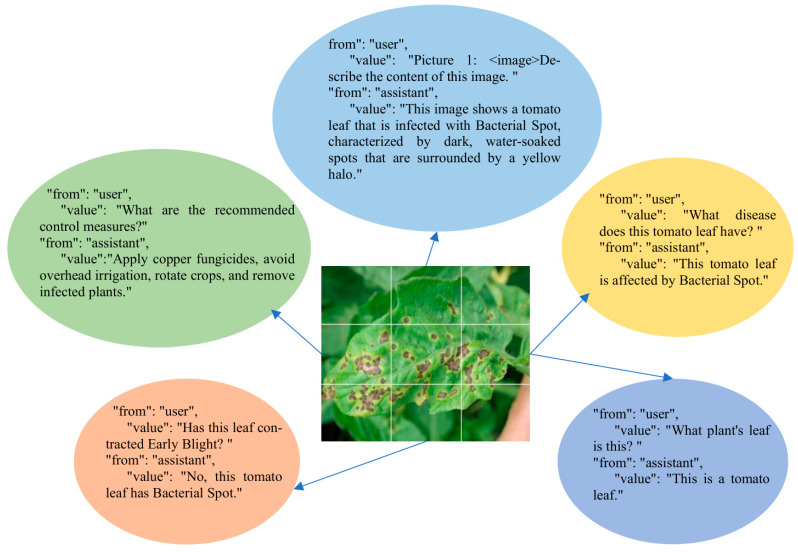
Example of multimodal instruction data.

The instruction texts used in this study were template-based rather than AI-generated. Specifically, the user-side prompts were constructed from a set of manually designed template families covering disease identification, pathological feature description, and control recommendations, and were then programmatically instantiated for each sample. In this way, the linguistic form of the instructions was standardized while still preserving diversity in task type and expression. The corresponding assistant responses were organized according to the disease label and associated descriptive information in the dataset, so that each image–text pair provided explicit and structured supervision for multimodal fine-tuning. A representative example is shown in Figure 8. Through the autoregressive generation mechanism of language models [51], this design aligns discrete visual features with semantically rich textual descriptions and provides dense multimodal supervision for model training.

In addition to the CDDM-derived instruction-tuning subsets, an approximately balanced cross-domain test set was constructed from the PlantVillage dataset to evaluate the generalization ability of the proposed model under distribution shift. The test set covered all 38 plant disease/health categories in PlantVillage, with 52–53 images sampled for each category, resulting in a total of 2000 test samples. The detailed class composition of this cross-domain test set is presented in Table 8.

It should be emphasized that this PlantVillage-based test set was not used for model training, LoRA parameter optimization, hyperparameter adjustment, or checkpoint selection. It was used only for the final cross-domain evaluation after model training was completed. This design ensured that the reported cross-domain performance reflected the model’s ability to transfer from the CDDM training domain to an independent PlantVillage test domain, rather than its ability to fit the training data distribution.

### 4.4. Dual-Side Collaborative LoRA Full-Link Fine-Tuning Method

#### 4.4.1. Low-Rank Adaptation (LoRA) Framework

A conventional full fine-tuning approach [52] initializes the model with pretrained weights, denoted by $\;\Phi_{0}$, and repeatedly updates them along the gradient direction to $\Phi_{0}+∆\Phi$, so as to maximize the conditional language modeling objective:

(1) $$\max_{\Phi }\sum_{\left(x,y\right)\in Z}\sum_{t=1}^{\left|y\right|}\log\left(P_{\Phi }\left(\mathrm{yt}|x,y<t\right)\right)$$

A major drawback of full fine-tuning is that an independent set of parameters must be learned for each downstream task. Given the large parameter scale of Qwen2.5-VL, conventional full fine-tuning is highly challenging and may even be impractical under limited computational resources. Moreover, Qwen2.5-VL was designed for general-purpose domains rather than crop disease recognition specifically, so its responses are not sufficiently precise without adaptation. To adapt a generic multimodal model to the crop disease domain under limited experimental conditions while avoiding the high computational cost and catastrophic forgetting risk associated with full fine-tuning, we adopt low-rank adaptation (LoRA) [45]. LoRA freezes most pretrained parameters and trains only a small number of injected low-rank matrices. By optimizing a rank-decomposed representation of weight updates in dense layers while keeping the pretrained weights unchanged, LoRA substantially reduces memory and computational requirements, allowing efficient customization of large models for specific tasks while retaining performance close to that of full-parameter fine-tuning.

**Figure 9:**
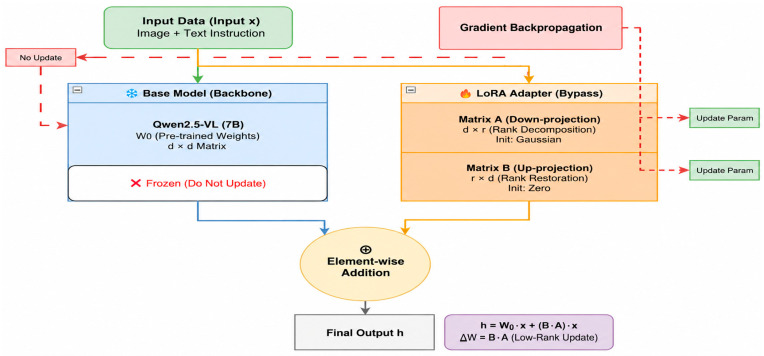
Workflow of LoRA fine-tuning.

As illustrated in Figure 9, the LoRA fine-tuning workflow introduces low-rank matrices A  and B  to decompose the weight-update increment ΔW into a low-rank form. During fine-tuning, the original pretrained backbone weight $W_{0}$ remains frozen, and only the reparameterized matrices A and B in the side branch are updated. During forward propagation, the hidden-layer output is formed by a linear combination of the original-weight path and the low-rank adaptation path. By constraining the parameter optimization space to a low-dimensional manifold, this strategy not only effectively mitigates the risk of catastrophic forgetting but also substantially reduces GPU memory usage and computational overhead, thereby improving training efficiency.

For the pretrained weight matrix $W_{0}\in R^{d\cdot d}$, the parameter update is represented through low-rank decomposition, with the update increment defined as $\Delta W=\frac{\alpha }{r}\mathrm{BA}$ The adapted weight after LoRA fine-tuning can be expressed as follows:

(2) $$h=W_{0}+\Delta \mathrm{Wx}=W_{0}x+\frac{\alpha }{r}\mathrm{BAx}$$

In this formulation, W denotes the frozen original pretrained weight of Qwen2.5-VL, which does not receive gradient updates, and ΔW denotes the increment produced by the LoRA branch. α is a scaling factor that controls the contribution of the low-rank update to the final output. It is typically treated as a constant, whereas the adaptation strength is regulated through the low-rank dimension r.

Our adoption of LoRA for Qwen2.5-VL is motivated by three considerations. First, from the perspective of computational efficiency, LoRA freezes the pretrained backbone and optimizes only low-rank factor matrices, thereby sharply reducing the number of trainable parameters and GPU memory usage. Second, in terms of model performance, prior studies have shown that LoRA can cut training parameters and GPU-memory requirements while achieving performance comparable to full-scale fine-tuning [34]. Third, LoRA offers outstanding deployment flexibility with no additional inference latency. Through reparameterization [53], adapter weights can be merged into the backbone at inference time, and different downstream tasks can be switched simply by replacing lightweight adapters rather than storing multiple full model replicas. This makes the proposed framework well suited to flexible edge deployment in agricultural scenarios.

#### 4.4.2. Dual-Side Injection into the Vision and Language Modules

The mainstream fine-tuning paradigm for multimodal large language models currently adopts a frozen vision encoder strategy, in which only the language model is updated while the pretrained visual feature extractor remains fixed. In generic applications, this approach can preserve broadly transferable visual features learned from large-scale datasets and reduce memory overhead considerably. However, when such a strategy is applied to a fine-grained vertical domain such as plant disease recognition, its limitations become evident. Plant disease diagnosis is a typical fine-grained visual task: the model must distinguish subtle differences in lesion color, texture, boundary, morphology, and spatial distribution. Generic visual encoders are biased toward high-level semantic representations of natural images and, when frozen, often fail to adapt to the domain-specific local feature distributions present in agricultural disease images. As a result, the extracted visual representations may not sufficiently characterize key pathological details, which in turn degrades subsequent cross-modal interaction and classification performance. Therefore, plant disease recognition requires a dedicated adaptation mechanism for fine-grained representation learning to improve both visual encoding and cross-modal alignment. From the perspective of parameter-efficient multimodal adaptation, previous LoRA-based methods mainly emphasize reducing optimization cost while preserving the general capabilities of pretrained backbones. However, in plant disease diagnosis, parameter efficiency alone is not sufficient. The key challenge lies in whether the adaptation mechanism can simultaneously improve fine-grained visual sensitivity and semantic-level reasoning. For this reason, our framework is designed not as a simple extension of LoRA to two modules, but as a collaborative adaptation strategy in which the visual branch is responsible for disease-specific perceptual refinement and the language branch is responsible for semantic reasoning and instruction compliance. This dual-side design distinguishes our method from conventional single-side LoRA or frozen-vision adaptation schemes.

To address this limitation, we propose a dual-side synergistic LoRA strategy. Unlike previous parameter-efficient multimodal adaptation methods, which typically adapt only the language model or retain a frozen vision encoder to preserve generic visual representations, our method partially unfreezes the vision encoder and injects trainable low-rank adapters into both the visual and language-generation modules, thereby constructing a fully trainable full-chain architecture. More importantly, the proposed framework is not merely distinguished by the placement of LoRA modules, but by the collaborative learning mechanism it enables: the visual branch is optimized for disease-specific perceptual refinement, while the language branch is optimized for semantic reasoning and multimodal instruction following. Under a unified autoregressive objective, this dual-side design allows visual perception and textual supervision to be jointly coupled during adaptation, which is particularly important for fine-grained plant disease diagnosis in complex field environments. This framework adopts end-to-end joint fine-tuning with LoRA. In contrast to the complex two-stage alignment procedures commonly used in multimodal models, such as contrastive learning, our approach directly achieves deep fusion between visual features and textual semantics through a single autoregressive objective. In this sense, the proposed framework differs from conventional LoRA-based multimodal tuning not only in parameter placement, but also in the learning mechanism by which fine-grained visual adaptation and language supervision are explicitly integrated.

The Qwen2.5-VL backbone consists of a Vision Transformer (ViT)-based vision encoder and a Transformer-decoder-based large language model, connected by a feature-compression (pooling) module and a multilayer perceptron (MLP) projection layer. To enable efficient domain adaptation while retaining pretrained knowledge, we do not fine-tune all parameters. Instead, LoRA is used to update only key weight matrices through low-rank decomposition. The vision encoder of Qwen2.5-VL is capable of handling dynamic resolutions, which ensures that subtle pathological features in the original high-resolution images are preserved as completely as possible, and allows an input image $W_{0}^{v}\in R^{d^{\mathrm{model}}\times d^{\mathrm{model}}}$ with an arbitrary aspect ratio to be converted into a variable-length visual sequence. To equip the model with domain-specific visual perception, we inject LoRA adapters into every layer of the vision encoder.

LoRA adapters are injected into the multi-head self-attention (MHSA) module of each layer in the vision encoder [54]. Let $W_{0}^{v}\in R^{d^{\mathrm{model}}\times d^{\mathrm{model}}}$ denote the frozen (Query) or (Value) projection weight matrix in the vision encoder. We constrain the parameter update ∆W to the product of two low-rank matrices, that is, $∆W\;=\;A_{v}B_{v}$, where $A_{v}\in R^{r\times d_{\mathrm{model}}}$ is initialized with a Gaussian distribution and the rank satisfies $r\ll d^{\mathrm{model}}$. The forward computation of the modified visual layer can then be expressed as follows:

(3) $$h=W_{0}x+\Delta \mathrm{Wx}=W_{0}x+\frac{\alpha }{r}(B_{v}A_{v}x)$$

In this formulation, x denotes the input visual hidden state and α is the scaling factor. By optimizing the parameter set $\theta_{v}\;=\;\{A_{v}B_{v}\},$ The vision encoder can adaptively adjust the attention-weight distribution according to downstream task requirements, thereby enabling the model to focus on key lesion regions already at the stage of pixel-level feature extraction.

Meanwhile, on the language-model side $E_{l}$, we likewise inject an independent LoRA parameter set, $\theta_{l}\;=\;\{A_{l}B_{l}\}$, into the self-attention layers. This part of the fine-tuning process is mainly responsible for high-level semantic reasoning, multimodal instruction following, and the generation of text responses that are consistent with human logic. The two parameter sets, $\theta_{v}$ and $\theta_{l}$, constitute the only trainable parameters in the proposed framework. During training, they operate collaboratively, with $\theta_{v}$ primarily responsible for “seeing the details” and $\theta_{l}$ for “understanding the logic.”

#### 4.4.3. Full-Chain Gradient Backpropagation and Task-Driven Perception

The core innovation of our framework lies in full-chain gradient backpropagation. The input sequence of the model is composed of visual features $E_{V}\left(I;\theta_{v}\right)$ and textual instruction embeddings $T_{\mathrm{in}}$ After being mapped through the projection layer, the joint embedding fed into the LLM can be expressed as follows:

(4) $$X_{\mathrm{joint}}\;=\;\left[P\left(E_{V}\left(I;\theta_{v}\right)\right);\;\mathrm{Embed}(T_{\mathrm{in}})\right]$$

**Figure 10:**
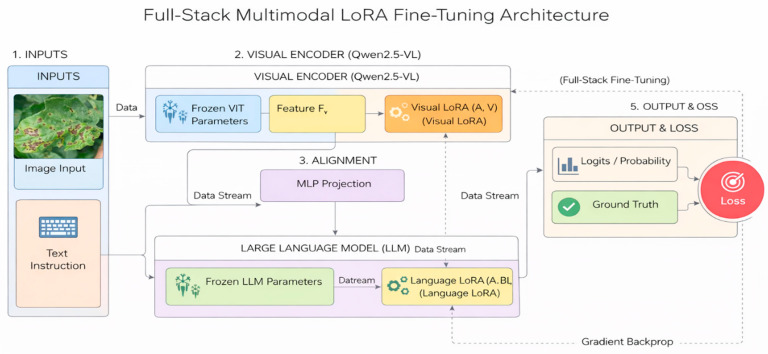
Full-chain fine-tuning framework.

Unlike the complex two-stage alignment strategies often used in multimodal models, such as contrastive learning, our framework directly fuses visual features and textual semantics through a single autoregressive objective. As illustrated in Figure 10, LoRA-based fine-tuning proceeds in three stages. In stage 1, the model processes the input image without forcibly resizing it to a fixed resolution. Instead, the dynamic-resolution mechanism of Qwen2.5-VL enables the ViT-based vision encoder to receive images while preserving their original aspect ratios and to divide them into patches, which are then converted into a visual feature sequence. This design preserves the fine-grained texture cues of plant diseases to the greatest extent possible. The extracted visual features are passed through an MLP projection layer, where a pooling operation is used to reduce the number of visual tokens, followed by a linear layer that maps the visual feature dimension into the textual embedding space of Qwen2.5. This step accomplishes the numerical transfer from visual signal to language signal.

In stage 2, we freeze most pretrained parameters in both the vision encoder and the language model and update only the injected LoRA modules. LoRA in the attention layers of the vision encoder promotes sensitivity to domain-specific disease features, whereas LoRA in the language model promotes the generation of domain-specific instruction formats and professional terminology. In stage 3, the model is optimized under a unified autoregressive generation task. The projected visual token sequence is directly concatenated with the textual instruction sequence to form a long multimodal input sequence. To preserve the two-dimensional structure of the image, the M-RoPE module of Qwen2.5-VL encodes temporal, height, and width information into the tokens. The concatenated sequence is then fed into the Qwen2.5 language model, which autoregressively predicts disease-description text conditioned on both the visual context and the textual instruction. The entire model is trained end-to-end with cross-entropy loss, and gradients flow from the language model through the MLP projection layer back into the vision encoder, thereby achieving visual–language alignment [55].

Compared with conventional fine-tuning that freezes the vision encoder, the proposed full-chain end-to-end strategy offers clear theoretical advantages. First, it reduces the risk of modality misalignment. Under frozen-encoder fine-tuning, the projection layer alone must map generic visual features into domain-specific semantics, which easily leads to overfitting of the projection layer. In our framework, by contrast, the vision encoder actively adjusts its feature distribution, making the mapping task of the projection layer smoother and more nearly linear.

Second, the framework realizes task-driven perception. Conventional visual encoders are task-agnostic and tend to treat all image features with equal importance. In our end-to-end setting, the gradient signals induced by task-relevant textual keywords effectively serve as attention guidance, training the vision encoder to suppress background noise and focus on visual regions that are highly relevant to the textual description. This mechanism can markedly improve robustness and accuracy in complex scene understanding.

## 5. Conclusions

This study proposes a dual-side synergistic low-rank adaptation framework built on Qwen2.5-VL for precise diagnosis and description generation of plant diseases in complex field environments. By partially unfreezing the vision encoder and establishing an end-to-end gradient backpropagation pathway, the framework achieves deep alignment between visual features and textual semantics. Experiments show that injecting LoRA adapters into both the vision encoder and the language model substantially improves the model’s ability to capture fine-grained disease characteristics. After training on the CDDM dataset, the model achieves 98.8% Top-1 accuracy in same-domain evaluation and 96.0% in cross-domain evaluation, markedly outperforming generic backbone models. Compared with models trained only on laboratory-style datasets such as PlantVillage, the proposed method shows substantially stronger resistance to background interference. Through multimodal joint training, the framework effectively mitigates dataset bias induced by a single background type. While maintaining high performance, the model also preserves favorable computational efficiency and interpretability, offering a practical technical route toward offline, real-time, expert-level interactive diagnostic assistants for smart agriculture.

Future work will focus on three directions. First, we will explore lightweight deployment through quantization, structured pruning, and related model-compression strategies so that the framework can be better adapted to low-power devices with limited computational resources. Second, we will further improve the model’s ability for rare disease recognition, especially for less common crops and long-tail disease categories, by investigating stronger few-shot learning and data-efficient adaptation strategies. Third, we will enhance model interpretability by introducing more transparent visual and multimodal evidence so that the diagnostic process can provide not only predictions but also more explicit and trustworthy explanations. In addition, we plan to integrate retrieval-augmented generation (RAG) [56] together with specialized knowledge graphs, thereby extending the system from single-disease diagnosis toward more advanced etiological reasoning and integrated disease-management recommendations.

## Figures and Tables

**Table 1:** Performance comparison of representative models for plant disease recognition under same-domain and cross-domain settings.

Method	Model Architecture	Training Set	Test Set	Evaluation Setting	Training Paradigm	Accuracy (%)
Mohanty et al. [14]	GoogLeNet(best model)	PlantVillage	Held-out PlantVillage test set	In-domain	Fully supervised	99.35
Mohanty et al. [14]	GoogLeNet(best model)	PlantVillage	External validation sets (dataset1/dataset2)	Cross-domain	Fully supervised	31.40/31.69
Qwen-VL-Chat [30]	Qwen-VL-Chat-7B	pretraining	Held-out CDDM test set (3000)	without agricultural adaptation	Backbone model	28.4
LLaVA-v1.5-7B [31]	ViT + LLaMA-7B	pretraining	Held-out CDDM test set (3000)	without agricultural adaptation	Backbone model	24.5
Qwen-VL-Chat-AG [29]	Qwen-VL-Chat-7B	CDDM137k	Held-out CDDM test set (3000)	In-domain	Fine-tuning	91.5
LLaVA-AG [29]	ViT + LLaMA-7B	CDDM137k	Held-out CDDM test set (3000)	In-domain	Fine-tuning	91.8
Ours	ViT + Qwen-VL-7B	CDDM50k	Held-out CDDM test set (3000)	In-domain	LoRA fine-tuning	98.8
Ours	ViT + Qwen-7B	CDDM50k	PlantVillage	Cross-domain	LoRA fine-tuning	96.0

**Table 2:** Ablation study of vision-side and language-side LoRA on plant disease diagnosis.

Setting	Vision-Side LoRA	Language-Side LoRA	In-Domain Top-1 (%)	Cross-Domain Top-1 (%)	Cross-Domain Macro -P (%)	Cross-Domain Macro -R (%)	Cross-Domain Macro -F1 (%)
Language-only	×	√	74.6	52.3	51.4	50.6	51.0
Vision-only	√	×	87.4	85.5	83.7	82.9	83.3
Dual-side (Ours)	√	√	98.8	96.0	95.4	94.8	95.1

Note: √ indicates that the corresponding module was used, whereas × indicates that it was not used.

**Table 3:** Representative misclassification examples and difficult but correctly recognized cases under cross-domain evaluation.

No.	Image	JSONL Row No.	Ground Truth	Prediction	Result	Possible Reason
1	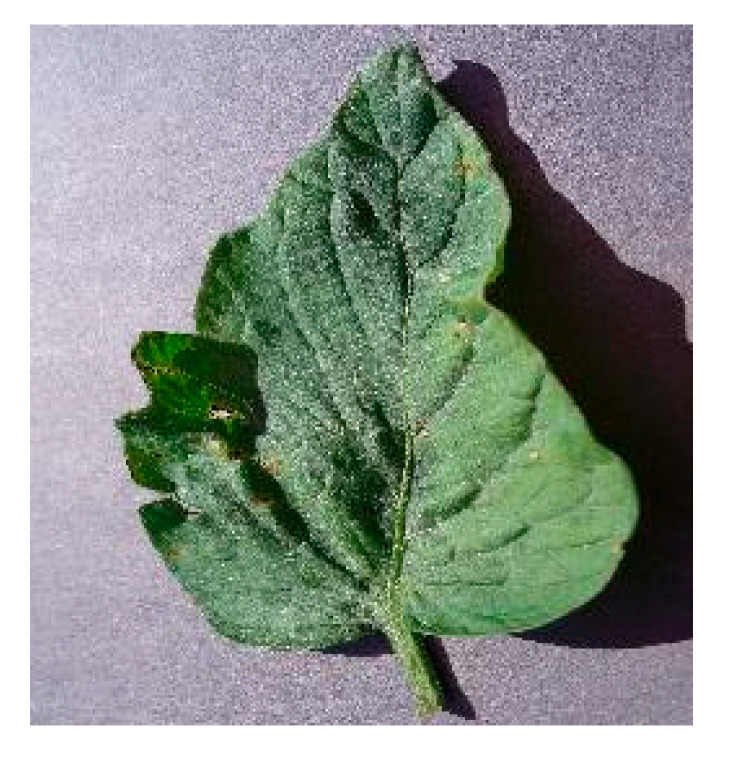	614	Tomato Target Spot	Bacterial Leaf Spot	Incorrect	The lesion pattern is characterized by small, densely distributed brown spots, whose boundaries and spatial distribution resemble those of bacterial leaf spot. As a result, the model is more easily influenced by local spot-density features and tends to confuse the two categories.
2	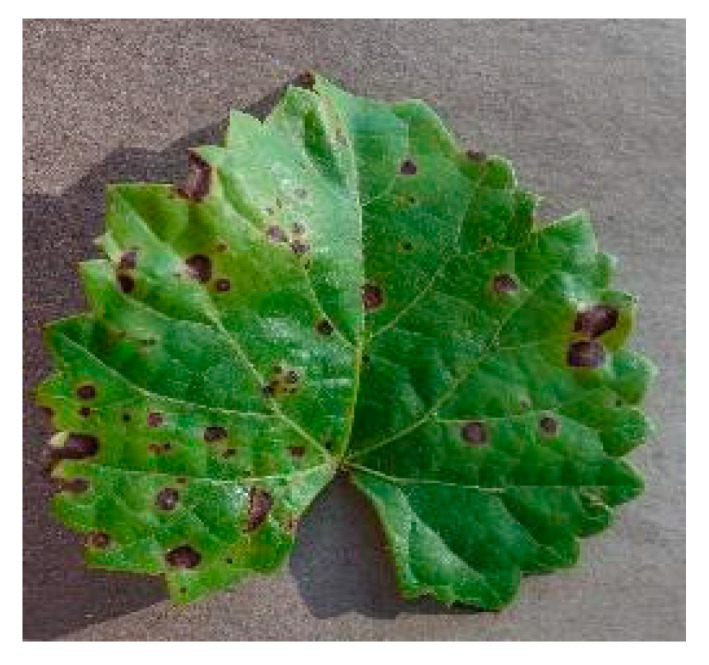	625	Grape Black Rot	Grape Brown Spot	Incorrect	These two diseases share a high degree of similarity in lesion color and necrotic appearance. When symptoms are relatively mild, the model may have greater difficulty capturing the darker necrotic center that is more typical of black rot.
3	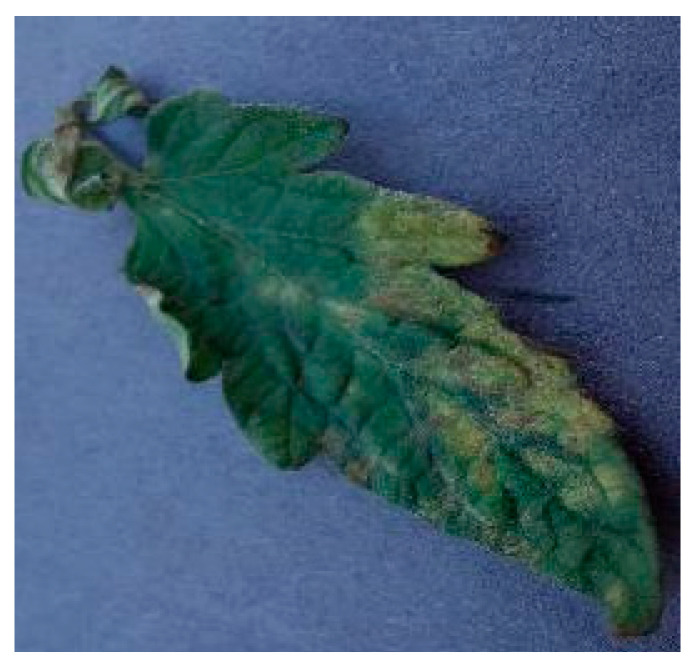	705	Tomato Leaf Mold	Tomato Target Spot	Incorrect	Both diseases may present as brown lesions on the leaf surface and exhibit similar local texture patterns and spot contours. Therefore, misclassification is more likely when the prediction relies primarily on local visual cues.
4	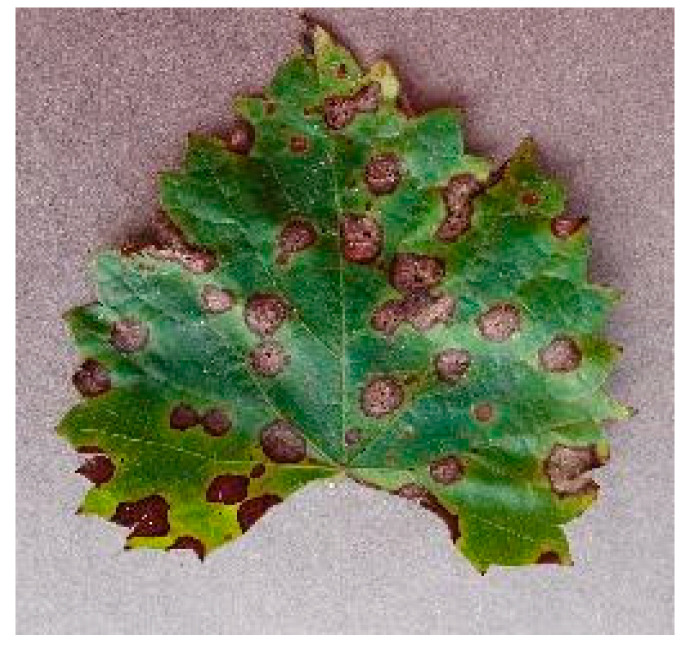	717	Grape Black Rot	Grape Black Rot	Correct	Although this sample is visually similar to grape brown spot in lesion color and morphology, the model correctly identified the more pronounced necrotic center and boundary pattern associated with grape black rot.
5	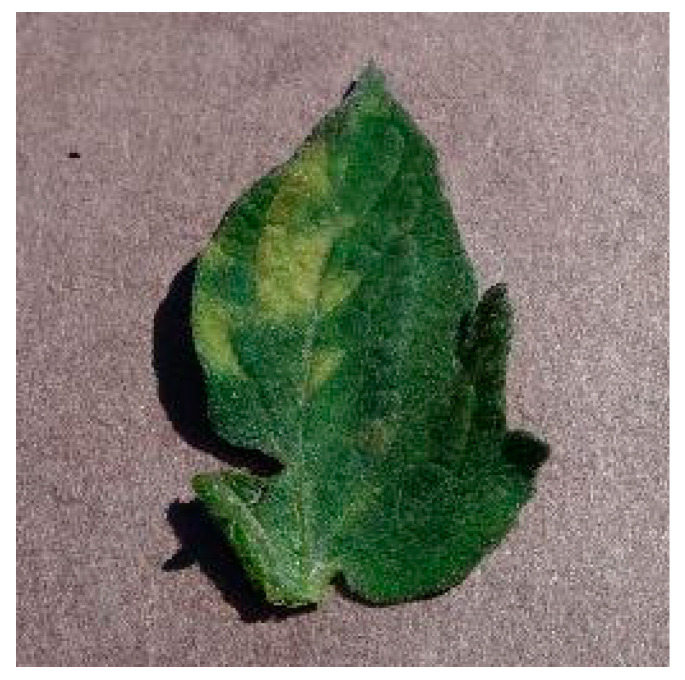	781	Tomato Leaf Mold	Tomato Leaf Mold	Correct	Although the symptoms show some similarity to Tomato Target Spot, the model still made the correct prediction by capturing differences in lesion distribution and texture.
6	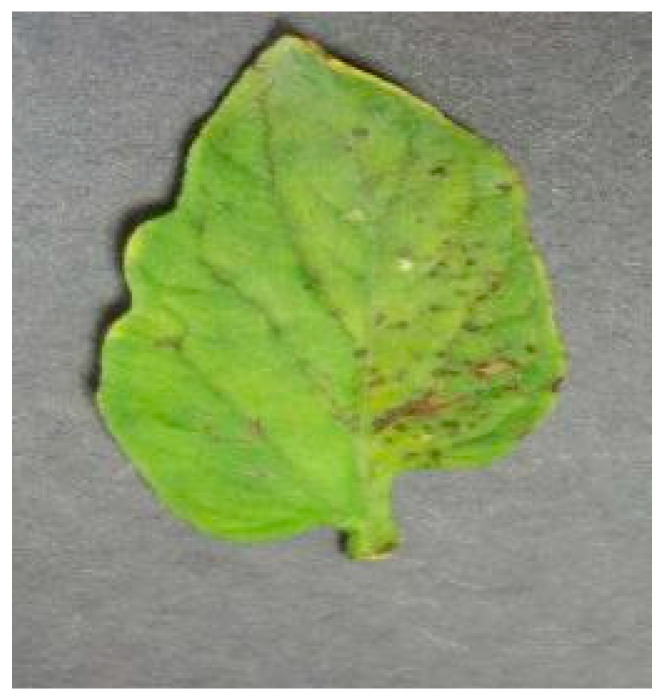	821	Tomato Bacterial Spot	Tomato Bacterial Spot	Correct	This sample overlaps with Tomato Target Spot in terms of small, densely distributed lesions; however, the model was still able to capture boundary and distribution characteristics more consistent with bacterial spot.

**Table 4:** Statistical stability analysis of the proposed dual-side framework over five random seeds.

Setting	Run 1	Run 2	Run 3	Run 4	Run 5	Mean ± Std
In-domain Top-1 (%)	98.8	97.9	98.7	98.5	98.6	98.50 ± 0.35
Cross-domain Top-1 (%)	96.0	95.2	95.8	95.6	95.9	95.70 ± 0.32

**Table 5:** Crop categories and disease classes in the PlantVillage dataset.

Class	Crop	Disease	Images	Class	Crop	Disease	Images
C01	Apple	Apple scab	630	C20	Bell pepper	Healthy	1478
C02		Black rot	622	C21	Potato	Early blight	1000
C03		Rust	275	C22		Healthy	152
C04		Healthy	1645	C23		Late blight	1000
C05	Blueberry	Healthy	1502	C24	Raspberry	Healthy	371
C06	Cherry	Healthy	854	C25	Soybean	Healthy	5090
C07		Powdery mildew	1052	C26	Squash	Powdery mildew	1855
C08	Corn	Gray spot	513	C27	Strawberry	Healthy	656
C09		Common rust	1192	C28		Leaf scorch	1109
C10		Healthy	1162	C29	Tomato	Bacterial spot	2127
C11		Northern leaf blight	985	C30		Early blight	1000
C12	Grape	Black rot	1180	C31		Healthy	1591
C13		Esca (black measles)	1383	C32		Late blight	1909
C14		Healthy	423	C33		Leaf mold	952
C15		Brown spot	1076	C34		Septoria leaf spot	1771
C16	Orange	Citrus greening	5507	C35		Spider mites	1676
C17	Peach	Bacterial spot	2297	C36		Target spot	1604
C18	Peach	Healthy	360	C37		Mosaic virus	373
C19	Bell pepper	Bacterial spot	997	C38		Yellow leaf curl virus	5357

**Table 6:** Crop categories and disease classes in the CDDM dataset.

Class	Crop	Disease	Images	Class	Crop	Disease	Images
C01	Apple	Alternaria blotch	5343	C31	Pumpkin	Powdery mildew	1835
C02		Black rot	621	C32	Raspberry	Healthy	371
C03		Brown spot	5655	C33	Rice	Bacterial leaf blight	1624
C04		Rust	275	C34		Rice blast	1520
C05		Frog eye leaf spot	3181	C35		Brown spot	1640
C06		Gray spot	4810	C36		Brown spot	80
C07		Healthy	6269	C37		Leaf smut	40
C08		Leaf rust	7554	C38		Tungro disease	1388
C09		Mosaic virus	4875	C39	Potato	Early blight	1000
C10		Powdery mildew	1184	C40		Healthy	152
C11		Apple scab	729	C41		Late blight	1000
C12	Bell pepper	Bacterial spot	997	C42	Tomato	Bacterial spot	5784
C13		Healthy	1478	C43		Early blight	4179
C14	Blueberry	Healthy	1502	C44		Healthy	5358
C15	Cherry	Healthy	854	C45		Late blight	5923
C16		Powdery mildew	1052	C46		Leaf mold	4529
C17	Corn	Healthy	1162	C47		Mosaic virus	3163
C18		Common rust	1192	C48		Powdery mildew	1256
C19		Gray spot	513	C49		Septoria leaf spot	5545
C20		Northern leaf blight	996	C50		Spider mites	3858
C21	Grape	Black rot	1180	C51		Target spot	3688
C22		Esca (black measles)	1383	C52		Yellow leaf curl virus	7967
C23		Healthy	423	C53	Wheat	Healthy	1524
C24		Brown spot	1076	C54		Leaf rust	1980
C25	Orange	Citrus greening	5507	C55		Loose smut	939
C26		Healthy	48	C56		Root rot	805
C27	Peach	Bacterial spot	2297	C57		Septoria tritici blotch	97
C28		Healthy	360	C58		Stem rust	376
C29	Strawberry	Healthy	456	C59		Stripe rust	208
C30		Leaf scorch	1109	C60	Soybean	Healthy	509

**Table 7:** Class composition and sample counts of the four sub-datasets.

Dataset		Dataset 1	Dataset 2	Dataset 3	Dataset 4	
Crop	Disease Class	1200	5868	27,146	50,841	Total
**Tomato**	Tomato Bacterial Spot	20	100	500	1000	1620
	Tomato Early Blight	20	100	500	1000	1620
**Apple**	Apple Alternaria Blotch	20	100	500	1000	1620
	Apple Black Rot	20	100	500	1000	1620
**Potato**	Potato Early Blight	20	100	500	1000	1620
	Potato Late Blight	20	100	500	1000	1620
	Potato Healthy	20	100	144	144	408
**Pepper Bell**	Bell Pepper Bacterial Spot	20	100	500	1000	1620
**Other crops**	Pepper Bell Healthy	20	100	500	1000	1620
	Cherry Powdery Mildew	20	100	500	986	1606
	Orange Citrus Greening	20	100	500	1000	1620
	Peach Bacterial Spot	20	100	500	1000	1620
	Pumpkin Powdery Mildew	20	100	500	1000	1620
	Strawberry Leaf Scorch	20	100	500	1000	1620
	…	…	…	…	…	…
Total	60 disease classes	**1200**	**5868**	**27** **,** **146**	**50** **,** **841**	**85** **,** **055**

**Table 8:** Composition of the balanced PlantVillage-based cross-domain test set.

Crop	Disease Class	2000
**Apple**	Apple scab	53
	Black rot	53
	Rust	53
**Grape**	Black rot	53
	Esca (black measles)	53
	Brown spot	53
	Healthy	53
**Potato**	Early blight	53
	Late blight	53
	Healthy	53
**Tomato**	Bacterial spot	53
	Leaf mold	53
	Target spot	53
**Other crops**	Cherry powdery mildew	53
	Strawberry leaf scorch	52
	…	…
**Total**	38 disease/health classes	2000

## Data Availability

The original contributions presented in this study are included in the article. Further inquiries can be directed to the corresponding author.
